# TikTok as a Source of Health Information and Misinformation for Young Women in the United States: Survey Study

**DOI:** 10.2196/54663

**Published:** 2024-05-21

**Authors:** Ciera E Kirkpatrick, LaRissa L Lawrie

**Affiliations:** 1 College of Journalism & Mass Communications University of Nebraska-Lincoln Lincoln, NE United States; 2 School of Journalism University of Missouri Columbia, MO United States

**Keywords:** credibility perceptions, health information, health misinformation, information seeking, misinformation perceptions, public health, social media, strategic communication, third-person effect, TikTok

## Abstract

**Background:**

TikTok is one of the most-used and fastest-growing social media platforms in the world, and recent reports indicate that it has become an increasingly popular source of news and information in the United States. These trends have important implications for public health because an abundance of health information exists on the platform. Women are among the largest group of TikTok users in the United States and may be especially affected by the dissemination of health information on TikTok. Prior research has shown that women are not only more likely to look for information on the internet but are also more likely to have their health-related behaviors and perceptions affected by their involvement with social media.

**Objective:**

We conducted a survey of young women in the United States to better understand their use of TikTok for health information as well as their perceptions of TikTok’s health information and health communication sources.

**Methods:**

A web-based survey of US women aged 18 to 29 years (N=1172) was conducted in April-May 2023. The sample was recruited from a Qualtrics research panel and 2 public universities in the United States.

**Results:**

The results indicate that the majority of young women in the United States who have used TikTok have obtained health information from the platform either intentionally (672/1026, 65.5%) or unintentionally (948/1026, 92.4%). Age (959/1026, 93.47%; *r*=0.30; *P*<.001), education (959/1026, 93.47%; ρ=0.10; *P*=.001), and TikTok intensity (ie, participants’ emotional connectedness to TikTok and TikTok’s integration into their daily lives; 959/1026, 93.47%; *r*=0.32; *P*<.001) were positively correlated with overall credibility perceptions of the health information. Nearly the entire sample reported that they think that misinformation is prevalent on TikTok to at least some extent (1007/1026, 98.15%), but a third-person effect was found because the young women reported that they believe that other people are more susceptible to health misinformation on TikTok than they personally are (*t*_1025_=21.16; *P*<.001). Both health professionals and general users were common sources of health information on TikTok: 93.08% (955/1026) of the participants indicated that they had obtained health information from a health professional, and 93.86% (963/1026) indicated that they had obtained health information from a general user. The respondents showed greater preference for health information from health professionals (vs general users; *t*_1025_=23.75; *P*<.001); the respondents also reported obtaining health information from health professionals more often than from general users (*t*_1025_=8.13; *P*<.001), and they were more likely to act on health information from health professionals (vs general users; *t*_1025_=12.74; *P*<.001).

**Conclusions:**

The findings suggest that health professionals and health communication scholars need to proactively consider using TikTok as a platform for disseminating health information to young women because young women are obtaining health information from TikTok and prefer information from health professionals.

## Introduction

### Background

As one of the most-used and fastest-growing social media platforms in the world, TikTok has drastically changed American culture [1,2]. The social media platform, which allows users to create and watch short-form videos ranging in length from 15 seconds to 10 minutes, has >150 million active users in the United States and is expected to reach 955 million users worldwide by 2025 [3,4]. This rapid popularity has caught the attention of health communication scholars and practitioners because the platform is a vehicle for finding and disseminating information, including health-related content [5,6]. According to the Pew Research Center, the number of adults in the United States who regularly get news from TikTok has more than tripled (from 3% in 2020 to 10% in 2022). Adults aged <30 years are the most likely group, with a third (32%) of adults aged 18 to 29 years saying that they regularly get their news from TikTok [7].

Americans turned to the internet to find health information during the COVID-19 pandemic, and medical professionals and health institutions met them on TikTok, delivering pandemic-related information and recommendations [5]. The presence of health-related content has extended beyond COVID-19–related information and includes a wide range of health topics such as cervical cancer screening, chronic pulmonary obstructive disease, diabetes, mental health, and more [8-11]. Studies examining the engagement with this content [10,11] suggest that users like finding health information on the platform. A recent survey of 2000 Americans conducted by the prescription savings company CharityRx found that 1 in 5 Americans turns to TikTok for advice before going to their physician. Of the participants belonging to Generation Z, specifically, 1 in 3 reported obtaining health information from TikTok [12].

TikTok’s concise video format encourages users to convey their message in a brief yet engaging way, while the relaxed atmosphere and the capacity to engage with viewers facilitate a more intimate and authentic form of communication [13]. These videos may be especially memorable (and thus influential) because viewers can retrieve both visual and verbal information that they have processed and stored while watching the videos [14]. Social media platforms have the power to spread credible, useful health information [8]. However, recent research has indicated that 1 in 5 TikTok videos likely contains misinformation [15], and fact-checking has been found to be uncommon on social media [16]. Consequently, as young women turn to TikTok for health information, they may encounter both the beneficial aspects and drawbacks of this accessible platform. For these reasons, we suggest the need for a better empirical understanding of the extent to which young women are obtaining health information on TikTok and their associated perceptions and behaviors related to the information they encounter.

### Objectives

In light of the popularity of TikTok, we conducted a survey of young women (assigned female sex at birth) in the United States to better understand their use of the social media platform for health information as well as their perceptions of the platform’s health information and related communication sources. We focus on women aged 18 to 29 years for this study because women make up the majority of TikTok users in the United States and because survey data have revealed that users aged 18 to 19 years and 20 to 29 years were the 2 largest age groups using TikTok during the time period that this study was conducted [17,18]. Furthermore, it has been suggested that women may be especially affected by health information on TikTok. Mainstream news programs (eg, Good Morning America on the ABC network) have anecdotally reported that many young women turn to TikTok for health information and that obstetrician-gynecologists and other physicians have developed TikTok brands specifically to reach these users [6]. According to prior research, young women’s involvement with social media significantly influences their perceptions and behaviors concerning their health [19]; women are more likely to look for health information, including via the internet [20,21]; and women tend to have a leading role in the majority of decisions for their families’ health [22].

Specifically, we first sought to explore how often young women are intentionally and unintentionally obtaining health information from TikTok and their top reasons for obtaining health information from the platform. Second, we explored their perceptions of credibility (ie, the perceived credibility of TikTok health information overall) and misinformation in relation to the health information they see on TikTok, as well as their frequency of verifying the health information they see. Within this, we asked questions about perceived susceptibility to health misinformation on TikTok to see if a third-person effect might exist. The third-person effect is a communication theory that suggests that people tend to perceive that messages in the media have a greater effect on other people than on themselves [23], which, in the context of misinformation on TikTok, could cause young women to underestimate the potential impact of misinformation on their own health-related decisions and behaviors. Third, we explored perceptions and behaviors related to the top 2 types of sources that share health information on TikTok (*health professionals* and *general users*). In terms of perceptions, we examined how often young women obtain information from these sources, how much they prefer to obtain information from these sources, and how credible they perceive the information from these sources to be (ie, the perceived credibility of TikTok health information from health professionals and the perceived credibility of TikTok health information from general users). For behaviors related to these source types, we examined whether the young women have acted on health information they obtained from these sources, their likelihood of acting on health information from these sources in the future, and their likelihood of fact-checking information from these sources. In exploring each of these 3 areas, we also examined whether the women’s age, highest level of education, and level of TikTok intensity (defined as their emotional connectedness to TikTok and TikTok’s integration into their daily lives) had a relationship with their use of TikTok as a source of health information.

Altogether, this study seeks to help both health communication researchers and practitioners by illuminating the role that TikTok plays in young women’s acquisition of health information in the United States.

## Methods

### Design and Sample

A web-based survey of US women (assigned female sex at birth) aged 18 to 29 years (N=1172) was conducted between April and May 2023. While the sample was focused on individuals who were assigned female sex at birth, we also asked about their gender identity (refer to the Results section). The sample was recruited using a Qualtrics research panel as well as convenience sampling at 2 public universities in the United States.

### Ethical Considerations

The institutional review boards at the University of Nebraska-Lincoln (IRB 20230122526EX) and the University of Missouri (IRB 2095651) approved the study. Respondents recruited via the Qualtrics panel were compensated in agreement with their Qualtrics contract, and the respondents recruited at the universities were compensated with course credit.

### Procedure

The study began with a web-based informed consent form that briefly explained the purpose of the study and gave the survey respondents information about the study’s investigator, the expected length of the survey, and how their data would be used (for the reporting of aggregate data) and stored (in a password-protected electronic format). Respondents then answered screening questions, and respondents who were not assigned female sex at birth and not aged 18 to 29 years were excluded from the survey. After passing the eligibility criteria, respondents were asked whether they had ever used TikTok to either watch or post videos. The respondents who had used TikTok were then asked about their average amount of use, whether they had ever intentionally used TikTok to look for advice or information about their health or health care (and whether they had done so in the past 3 months), and whether they had ever unintentionally been exposed to health information on TikTok. From here, the questions they saw depended upon whether they had ever seen health information (intentionally or unintentionally) on TikTok.

All respondents who had ever used TikTok (n*=*1026) responded to items measuring reasons for health-related TikTok use; the perceptions of health misinformation on TikTok; the use of, and preference for, particular sources (health professionals and general users) of health information on TikTok; the perceived credibility of health information from health professionals and general users on TikTok; the likelihood of acting on health information obtained from health professionals and general users on TikTok; and the likelihood of fact-checking health information from health professionals and general users on TikTok.

Respondents who had seen health information on TikTok (n*=*959) additionally responded to items measuring their perceived credibility of the health information they have seen overall on TikTok and their verification of the health information they have seen on TikTok. Respondents who had ever received health information on TikTok from either source of interest (health professionals or general users) were also asked about whether they had acted on health information from these sources.

Finally, all participants (N*=*1172) responded to items measuring TikTok intensity (ie, their emotional connectedness to TikTok and TikTok’s integration into their daily lives) and answered demographic questions, including their highest level of education, race, and ethnicity.

### Measures

#### Using TikTok as a Health Information Source

##### Frequency of Use

Respondents were asked whether they had ever used TikTok (either to watch or to post videos). The respondents who had used TikTok were asked to indicate their average amount of use, using the following options: less than once a month, once a month, once a week, a few times a week, once a day, more often than once a day. The respondents who had ever used TikTok were also asked whether they had ever used TikTok to look for advice or information about their health or health care (selecting *yes* or *no*). Those who selected *yes* were asked how often they *intentionally* use TikTok to obtain health information, and all respondents who had ever used TikTok were asked how often they unintentionally obtain health information on TikTok (hourly, daily, weekly, monthly, less often, or not at all).

##### Reasons for Health-Related TikTok Use

Respondents indicated their reasons for health-related TikTok use by indicating their level of agreement (ranging from 1=*strongly disagree* to 7=*strongly agree*) with 10 different statements (eg, “I like to get health information from TikTok because it can help me to maintain a healthy lifestyle”) adapted from prior research [24-26] (refer to the Results section).

#### Credibility, Misinformation, and Verification of Health Information on TikTok

##### Perceived Credibility of TikTok Health Information Overall

Using the 7-point scale (ranging from 1=*not at all* to 7=*extremely*) for media credibility developed by Flanagin and Metzger [27], respondents rated how believable, accurate, trustworthy, biased (reverse coded), and complete they perceive health information on TikTok, overall, to be. Specifically, the respondents were asked, “To what degree do you rate the health information provided on TikTok?” The 5 items were averaged to create a perceived credibility score for each respondent (mean 4.48, SD 1.28; Cronbach α=0.90).

##### Misinformation Perceptions

For the perceptions of misinformation, respondents were asked to indicate on a 7-point scale how prevalent they think health misinformation is on TikTok (ranging from 1=*not prevalent at all* to 7=*very prevalent*; mean 5.14, SD 1.42), how serious they think the impact of health misinformation on TikTok is (ranging from 1=*not serious at all* to 7=*very serious*; mean 5.57, SD 1.46), and how susceptible they think they are to the influence of health misinformation on TikTok (ranging from 1=*not susceptible at all* to 7=*very susceptible*; mean 4.07, SD 1.75; adapted from the study by Chang [28]). In addition, to explore the possibility of a third-person effect, the respondents were asked to indicate how susceptible (ranging from 1=*not susceptible at all* to 7=*very susceptible*) they think others (eg, the public; mean 5.26, SD 1.46) are to the influence of health misinformation on TikTok (adapted from the study by van der Meer et al [23]). Participants were also asked about their self-perceived direct experience with health information on TikTok with the following item (adapted from the study by Chang [28]): “Have you encountered health misinformation on TikTok in the past? (*yes*, *no, or unsure*).”

##### Verification of Health Information on TikTok

Using a scale adapted from the study by Flanagin and Metzger [27], respondents indicated on a 7-point scale (ranging from 1=*never* to 7=*always*) how often they performed 6 different verification behaviors (eg, “Check to see if the information is current”) when seeing health information on TikTok. The scores for the 6 items were averaged to create a *verification of TikTok health information* score for each respondent (mean 4.83, SD 1.53; Cronbach α=0.92).

#### Health Professionals and General Users as Sources of Health Information on TikTok

##### Source Preferences

Using a 7-point scale (ranging from 1=*not at all* to 7=*very often*), respondents indicated how often they obtain health information from health professionals on TikTok (mean 5.04, SD 1.83) and how often they obtain health information from general users on TikTok (mean 4.55, SD 1.89). In addition, using a 7-point scale (ranging from 1=*don’t prefer them at all* to 7=*prefer them a lot*), respondents were asked to indicate how much they prefer to obtain health information from health professionals on TikTok (mean 5.65, SD 1.75) and how much they prefer to obtain health information from general users on TikTok (mean 4.08, SD 1.96).

##### Perceived Credibility of TikTok Health Information From Health Professionals and General Users

In addition to measuring the respondents’ perceived credibility of TikTok health information *overall*, we also measured the respondents’ perceived credibility of the 2 sources of interest (health professionals and general users) using the 7-point scale for media credibility developed by Flanagin and Metzger [27]. Specifically, we asked, “To what degree do you rate the health information provided by *health professionals* (eg, a doctor or nurse) on TikTok?” and “To what degree do you rate the health information provided by *general users* (someone like you) on TikTok?” Respondents rated how believable, accurate, trustworthy, biased (reverse coded), and complete they believe the health information on TikTok to be from each of these sources. The scores for each of the 5 items were averaged for each of the source types such that each respondent had a score for the perceived credibility of TikTok health information from health professionals (mean 5.16, SD 1.18; Cronbach α=0.90) and the perceived credibility of TikTok health information from general users (mean 3.95, SD 1.54; Cronbach α=0.94).

##### Acting on Health Information

Respondents’ likelihood of acting on health information was measured with items adapted from the study by Hu and Shyam Sundar [29]. Using a 7-point scale (ranging from 1=*not at all likely* to 7=*extremely likely*), respondents indicated how likely they are to act on health information from a health professional on TikTok (mean 4.50, SD 1.79) and from a general user on TikTok (mean 3.96, SD 1.89). Respondents were also asked whether they ever have acted on health information provided on TikTok by a health professional or general user.

##### Fact-Checking Information

Respondents were asked, on a scale ranging from 1=*not at all likely* to 7=*very likely*, to rate how likely they are to fact-check health information on TikTok from a health professional (mean 4.88, SD 1.80) and a general user (mean 5.37, SD 1.83).

#### Audience Characteristics

##### TikTok Intensity

Scores for TikTok intensity were created using an adapted form of the scale for Facebook intensity developed by Ellison et al [30]. The scale was created to measure how emotionally connected participants are to the social media platform as well as the extent to which the platform is part of their everyday lives. This TikTok-modified version of the scale (ranging from 1=*strongly disagree* to 7=*strongly agree*) asked respondents to rate their agreement with the following six items: (1) “TikTok is part of my everyday life,” (2) “I am proud to tell people I’m on TikTok,” (3) “TikTok has become part of my daily routine,” (4) “I feel out of touch when I haven’t logged into TikTok for a while,” (5) “I feel I am part of the TikTok community,” and (6) “I would be sorry if TikTok shut down.” The scores of the 6 items were averaged to create TikTok intensity scores for each respondent who had reported ever having used TikTok (mean 4.91, SD 1.49; Cronbach α=0.90).

##### Survey Questionnaire and Descriptive Statistics

The full survey questionnaire and descriptive statistics for each variable across the student and Qualtrics samples can be found in Multimedia Appendix 1 and Multimedia Appendix 2, respectively.

### Data Analysis

Statistical analyses were performed using SPSS software (version 29.0; IBM Corp). Descriptive analyses were conducted to describe the respondents’ frequency of TikTok use, frequency of intentional and unintentional exposure to health information on TikTok, reasons for health-related TikTok use, beliefs about encountering health misinformation, perceptions of misinformation on TikTok, frequency of performing verification behaviors on TikTok, frequency and preferences related to obtaining health information from health professionals and general users on TikTok, and frequency of acting on TikTok health information.

Bivariate correlational analyses were conducted to examine the relationships that credibility perceptions (the perceived credibility of TikTok health information overall, the perceived credibility of TikTok health information from health professionals, and the perceived credibility of TikTok health information from general users), misinformation perceptions, verification behaviors, the likelihood of acting on TikTok health information, and the likelihood of fact-checking TikTok health information had with the respondents’ age, highest level of education, and level of TikTok intensity. In addition, bivariate correlational analyses were conducted to examine the relationship between respondents’ perceived credibility of TikTok health information overall and their likelihood of acting on the health information. For these correlational analyses, a correlation was considered weak if the correlation coefficient was between −0.4 and 0.4. A correlation was considered moderate if the correlation coefficient was between −0.8 and −0.4 or between 0.4 and 0.8. A correlation was considered strong if the correlation coefficient was between −1 and −0.8 or between 0.8 and 1.

Finally, paired samples *t* tests (2-tailed) were conducted to observe the statistical differences between respondents’ perceived susceptibility of health misinformation on TikTok for themselves versus for others, frequency of obtaining health information on TikTok from health professionals versus general users, preference for obtaining health information on TikTok from health professionals versus general users, perceived credibility of TikTok health information from health professionals versus general users, likelihood of acting on TikTok health information from health professionals versus general users, and likelihood of fact-checking TikTok health information from a health professional versus a general user.

## Results

### Overview

A total of 1172 qualified responses were collected, with the average age of the sample being 22.82 (SD 3.15) years. A little more than half of the participants came from the Qualtrics panel (636/1172, 54.27%), and the rest were recruited through the universities (536/1172, 45.73%). The majority of the sample identified as White (910/1172, 77.65%), and most of the sample reported having used TikTok (1026/1172, 87.54%). Approximately half of the participants reported using TikTok more often than once a day (615/1172, 52.47%). Further demographic information is included in Table 1, and the full list of demographic questions can be found in Multimedia Appendix 1.

**Table 1:** Respondent demographics (N=1172).

Characteristics		Participants, n (%)
**Race**		
	American Indian or Alaska Native	3 (0.26)
	Asian	16 (1.37)
	Black or African American	117 (9.98)
	Native Hawaiian or Pacific Islander	57 (4.86)
	White	910 (77.65)
	Other	53 (4.52)
	Prefer not to answer	16 (1.37)
**Ethnicity**		
	Cuban	13 (1.11)
	Mexican, Mexican American, or Chicana	109 (9.3)
	Puerto Rican	11 (9.39)
	Other Spanish, Hispanic, or Latina	98 (8.36)
	Not Spanish, Hispanic, or Latina	919 (78.41)
	Prefer not to answer	22 (1.88)
**Education**		
	Less than high school	17 (1.45)
	High school graduate or equivalent (eg, GED^a^)	207 (17.66)
	Some college	509 (43.43)
	2-year degree	90 (7.68)
	4-year degree	224 (19.11)
	Professional or master’s degree	68 (5.8)
	Doctorate	57 (4.86)
**Self-identified gender**		
	Woman	1154 (98.46)
	Transgender	4 (0.34)
	Nonbinary	11 (0.94)
	Gender fluid	2 (0.17)
	Other	1 (0.09)
**Frequency of TikTok use**		
	Never	146 (12.46)
	Less than once a month	58 (4.95)
	Once a month	50 (4.27)
	Once a week	60 (5.12)
	A few times a week	108 (9.22)
	Once a day	135 (11.52)
	More often than once a day	615 (52.47)

^a^GED: General Educational Development test.

### Using TikTok as a Health Information Source

#### Frequency of Use

Of the 1026 respondents who had used TikTok before, 672 (65.5%) reported that they had *intentionally* used TikTok to look for advice or information about their health or health care, while 948 (92.4%) reported that they had *unintentionally* received health information or advice on TikTok. Of the 1026 respondents who had ever used TikTok, 582 (56.73%) reported having intentionally used TikTok to look for advice or information about their health or health care in the last 3 months. A breakdown of the frequency of intentional and unintentional exposure to health information on TikTok is provided in Table 2.

**Table 2:** Frequency of using TikTok intentionally and unintentionally as a source of health information among respondents who had ever used TikTok (n=1026).

Questions and responses			Participants, n (%)
**Frequency of intentional use of TikTok to obtain health information**			
	Hourly	29 (2.83)	
	Daily	108 (10.53)	
	Weekly	143 (13.94)	
	Monthly	168 (16.37)	
	Less often	190 (18.52)	
	Not at all	388 (37.82)	
**Frequency of unintentional exposure to health information on TikTok**			
	Hourly	38 (3.7)	
	Daily	232 (22.61)	
	Weekly	363 (35.38)	
	Monthly	172 (16.76)	
	Less often	143 (13.94)	
	Not at all	78 (7.6)	

#### Reasons for Health-Related TikTok Use

Of the 10 reasons presented for health-related TikTok use, obtaining advice from others with the same disease or health condition (mean 5.29, SD 1.54), receiving social support from others (mean 5.29, SD 1.57), and gaining knowledge about a disease they had been diagnosed with (mean 5.01, SD 1.59) were the most agreed upon reasons. The least agreed upon reason for health-related TikTok use was communicating with physicians (mean 4.06, SD 1.86). Table 3 shows the mean and SD of the level of agreement for each of the 10 reasons.

**Table 3:** Reasons for health-related TikTok use among respondents who had ever used TikTok (n=1026).

I like to get health information from TikTok because...	Level of agreement^a^, mean (SD)
It can help me to maintain a healthy lifestyle.	4.92 (1.60)
It can help me determine whether I need to see a doctor.	4.81 (1.67)
It can provide me with more information after I’ve seen my doctor.	4.65 (1.72)
It can help me find different options for treatment or maintenance of my health condition(s).	4.79 (1.65)
I can gain knowledge about a disease I’ve been diagnosed with.	5.01 (1.59)
I can obtain advice from other patients with the same disease or health condition as me.	5.29 (1.54)
I can receive social support from others.	5.29 (1.57)
I can communicate with physicians.	4.06 (1.86)
I can interact in real time with TikTok users.	4.59 (1.81)
I can obtain immediate health information and make use of it.	4.53 (1.71)

^a^Respondents indicated their level of agreement using a 7-point scale (ranging from 1=*strongly disagree* to 7=*strongly agree*).

### Credibility, Misinformation, and Verification of Health Information on TikTok

#### Perceived Credibility of TikTok Health Information Overall

With a mean of 4.48 (SD 1.28) on a 7-point scale, the credibility perceptions were moderate. A positive correlation was found (959/1026, 93.47%; *r*=0.30; *P*<.001) between the perceived credibility of health information on TikTok overall and the respondents’ age. Older participants tended to perceive the content as more credible. A positive correlation was also found (959/1026, 93.47%; ρ=0.10; *P*=.001) between the perceived credibility of health information on TikTok overall and the respondents’ highest level of education, with respondents with greater education tending to rate the health information on TikTok as more credible. There was a positive correlation (959/1026, 93.47%; r=0.32; *P*<.001) between TikTok intensity and the perceived credibility of TikTok health information overall. Respondents with higher TikTok intensity scores tended to have greater perceived credibility of health information on TikTok overall.

#### Misinformation Perceptions

Approximately half (563/1026, 54.87%) of the respondents who had used TikTok indicated that they believe that they have personally encountered health misinformation on the platform at some point, and only 1.85% (19/1026) of the sample stated that they think that health misinformation is not prevalent at all on TikTok. Table 4 shows the levels of perceived prevalence, severity, and susceptibility of health misinformation on TikTok reported by the respondents who had used TikTok.

There was a weak but significant negative correlation between age and the perceived seriousness of health misinformation on social media (1026/1172, 87.54%; *r*=−0.07; *P*=.04). There was not a significant relationship between age and perceived prevalence (1026/1172, 87.54%; *r*=−0.06; *P*=.05) or perceived susceptibility (1026/1172, 87.54%; *r*=−0.02; *P*=.50). There was not a significant relationship between the respondents’ highest level of education and the perceived prevalence (1026/1172, 87.54%; ρ=−0.008; *P*=.81), the perceived seriousness (1026/1172, 87.54%; ρ=0.01; *P*=.66), or the perceived susceptibility of health misinformation on TikTok (1026/1172, 87.54%; ρ=0.04; *P*=.17).

There was a weak but significant positive relationship between respondents’ TikTok intensity scores and perceived seriousness (1026/1172, 87.54%; ρ=0.07; *P*=.02) and perceived susceptibility (1026/1172, 87.54%; ρ=0.07, *P*=.02) such that respondents with high TikTok intensity scores tended to perceive greater seriousness and susceptibility of health misinformation on TikTok. There was not a significant relationship between TikTok intensity scores and the perceived prevalence of health misinformation on TikTok (1026/1172, 87.54%; ρ=0.03; *P*=.29).

The results also showed that respondents perceive other people (mean 5.26, SD 1.46) as more susceptible to health misinformation on TikTok than they personally are (mean 4.07, SD 1.75; *t*_1025_=21.16; *P*<.001).

**Table 4:** Perceived prevalence, seriousness, and susceptibility of health misinformation on TikTok among respondents who had ever used TikTok (n=1026).

Questions	Level of agreement^a^, mean (SD)
How prevalent is health misinformation on TikTok?	5.14 (1.42)
How serious do you think the impact of health misinformation on TikTok is?	5.57 (1.46)
How susceptible are you to the influence of health misinformation on TikTok?	4.07 (1.75)
How susceptible are other people to the influence of health misinformation on TikTok?	5.26 (1.46)

^a^Respondents indicated their level of agreement using a 7-point scale (ranging from 1=*not at all prevalent, serious, or susceptible to 7=very prevalent, serious, or susceptible*).

#### Verification of Health Information on TikTok

The most frequently used form of verification was considering whether the information represented was opinion or fact and the least frequently used form of verification was verifying the TikTok users’ qualifications or credentials. Table 5 shows the respondents’ frequency of each verification behavior.

There was a weak but significant positive correlation found between the respondents’ age and their likelihood of verifying TikTok health information (959/1026, 93.47%; *r*=0.20; *P*<.001) as well as between the respondents’ highest level of education and their likelihood of verifying TikTok health information (959/1026, 93.47%; ρ=0.09; *P*<.001). A weak but significant positive relationship existed between respondents’ TikTok intensity scores and their likelihood of verifying TikTok health information (959/1026, 93.47%; *r*=0.21; *P*<.001). Participants with high TikTok intensity scores were more likely to verify the health information.

**Table 5:** Verification of TikTok health information among participants who had seen health information on TikTok (n=959).

Verification behavior	Participant response^a^						
	Never, n (%)	Almost never, n (%)	Rarely, n (%)	About half of the time, n (%)	Most of the time, n (%)	Almost always, n (%)	Always, n (%)
Verify the TikTok users’ qualifications or credentials	121 (12.6)	68 (7.1)	89 (9.3)	131 (13.7)	193 (20.1)	178 (18.6)	179 (18.7)
Consider the TikTok users’ goals and objectives for posting information on the web	74 (7.7)	87 (9.1)	70 (7.3)	165 (17.2)	208 (21.7)	199 (20.8)	156 (16.3)
Check to see whether the information is current	72 (7.5)	61 (6.4)	66 (6.9)	143 (14.9)	223 (23.3)	198 (20.7)	196 (20.4)
Seek out other sources to validate the information	63 (6.6)	52 (5.4)	76 (7.9)	121 (12.6)	200 (20.9)	221 (23)	226 (23.6)
Consider whether the information represented is opinion or fact	53 (5.5)	50 (5.2)	49 (5.1)	125 (13)	199 (20.8)	251 (26.2)	232 (24.2)
Check to see that the information is complete and comprehensive	73 (7.6)	55 (5.7)	65 (6.8)	148 (15.4)	193 (20.1)	225 (23.5)	200 (20.9)

^a^Participants were asked to indicate how often they perform each of the 6 different behaviors when seeing health information on TikTok.

### Health Professionals and General Users as Sources of Health Information on TikTok

#### Source Preferences

Of the respondents who had ever used TikTok, 93.08% (955/1026) indicated that they had obtained health information from a health professional on the platform, while 93.86% (963/1026) indicated that they had obtained health information from a general user on the platform. That said, respondents reported obtaining health information from health professionals on TikTok (mean 5.04, SD 1.83) significantly more often than they obtain health information from general users on TikTok (mean 4.55, SD 1.89; *t*_1025_=8.13; *P*<.001). This was in line with their preferences for health information sources because the respondents’ preference for obtaining health information from health professionals (mean 5.65, SD 1.75) was significantly greater than their preference for obtaining health information from general users (mean 4.08, SD 1.96; *t*_1025_=23.75; *P*<.001).

#### Perceived Credibility of TikTok Health Information From Health Professionals and General Users

Respondents perceived health information from health professionals on TikTok (mean 5.16, SD 1.18) to be significantly more credible than health information provided by general users on TikTok (mean 3.95, SD 1.54; *t*_958_=26.737; *P*<.001).

#### Acting on Health Information

Of the respondents who had received health information from a health professional on TikTok, 43.35% (414/955) reported that they had acted on health information they obtained from a health professional on TikTok. In comparison, 37.8% (364/963) of the respondents who had received health information from a general user on TikTok reported that they had acted on health information they obtained on TikTok from a general user. When asked about their likelihood of acting on health information on TikTok in the future, the respondents’ likelihood of acting on health information from a health professional on TikTok (mean 4.50, SD 1.79) was significantly higher than their likelihood of acting on health information from a general user on TikTok (mean 3.96, SD 1.89; *t*_1025_=12.74; *P*<.001).

Likewise, the respondents’ perceived credibility of TikTok health information overall was positively correlated with their likelihood of acting on the health information. Respondents who perceived health information on TikTok overall as credible were more likely to act on health information they obtained from a health professional on TikTok (959/1026, 93.47%; *r*=0.47; *P*<.001) than from a general user on TikTok (959/1026, 93.47%; *r*=0.53; *P*<.001).

Age was weakly positively correlated with the respondents’ likelihood of acting on health advice found on TikTok both from a health professional (1026/1172, 87.54%; *r*=0.11; *P*<.001) and from a general user (1026/1172, 87.54%; *r*=0.23; *P*<.001). Likewise, education was weakly positively correlated with their likelihood of acting on health information found on TikTok both from a health professional (1026/1172, 87.54%; ρ=0.09; *P*=.003) and from a general user (1026/1172, 87.54%; ρ=0.08; *P*=.01).

There was a significant positive relationship between TikTok intensity and the respondents’ likelihood of acting on health information from health professionals on TikTok (1026/1172, 87.54%; *r*=0.41; *P*<.001). Likewise, there was a significant positive relationship between TikTok intensity and their likelihood of acting on health information from general users on TikTok (1026/1172, 87.54%; *r*=0.39; *P*<.001). Respondents with higher TikTok intensity scores tended to be more likely to act on health information from both health professionals and general users on TikTok.

#### Fact-Checking Information

There was a statistically significant difference in the respondents’ likelihood of fact-checking health information on TikTok from a health professional versus a general user. The likelihood of fact-checking health information from a general user (mean 5.37, SD 1.83) was higher than the likelihood of fact-checking health information from a health professional (mean 4.88, SD 1.80; *t*_1025_=9.71; *P*<.001).

Age was weakly positively correlated with the respondents’ likelihood of fact-checking TikTok health information both from a health professional (1026/1172, 87.54%; *r*=0.18; *P*<.001) and from a general user (1026/1172, 87.54%; *r*=0.07; *P*=.02). Education was also weakly positively correlated with their likelihood of fact-checking TikTok health information from a health professional (1026/1172, 87.54%; ρ=0.11; *P*<.001), but education was not correlated with their likelihood of fact-checking TikTok health information for a general user (1026/1172, 87.54%; ρ=0.04; *P*=.26).

There was a significant but weak positive relationship between TikTok intensity and the respondents’ likelihood of fact-checking TikTok health information from both health professionals (1026/1172, 87.54%; *r*=0.07; *P*=.03) and general users on TikTok (1026/1172, 87.54%; *r*=0.10; *P*=.002). Respondents with higher TikTok intensity scores tended to be more likely to fact-check health information from both health professionals and general users on TikTok.

## Discussion

### Principal Findings

TikTok has generated substantial attention due to recent reports suggesting its emergence as a significant source of information for many Americans [7]. For some users, TikTok has replaced traditional news networks as well as widely used search engines such as Google [31]. Given this emergence of TikTok as an information source and the presence of health information available on the platform [5,6], we surveyed 1172 women aged 18 to 29 years to understand their use of TikTok as a source of health information. Of the 1172 respondents, 1026 (87.54%) had used TikTok in some capacity.

The findings provide evidence that TikTok has become a source of health information for young women in the United States. More than half of the respondents who had ever used TikTok (672/1026, 65.5%) reported that they had intentionally used TikTok to look for advice or information about their health or health care, and nearly the entire sample of TikTok users (948/1026, 92.4%) reported having unintentionally obtained health information on TikTok. The popularity of health-related hashtags on TikTok (eg, as of November 2023, #medicaltiktok and #healthtok had 7.6 billion and 2.4 billion views, respectively) has illuminated some of TikTok’s popularity as a commonly searched platform for information related to health, but the findings of this study provide a greater empirical understanding of the extent to which young women actually obtain health information from the platform.

TikTok’s popularity as a source of health information may, in part, be the result of how technology has influenced human beings’ desire for immediate information. Rather than having to wait for a physician’s appointment to ask about one’s symptoms or health condition, one can take to the internet (eg, TikTok) and find related information in a matter of minutes [12]. This phenomenon of individuals seeking immediate information has important implications for health professionals. By knowing that individuals turn to platforms such as TikTok to find health information, health professionals can proactively create content so that credible health information is available when users go to find it. Social media platforms were heavily relied upon for health information during the COVID-19 pandemic [16], and since then, social media, and TikTok specifically, have been recommended as a tool for health promotion [5,32,33]. Given that TikTok is easily accessible and allows anyone to consume information without judgment, it may especially be helpful for populations with barriers to care and for communicating about taboo or stigmatized topics that users may be less comfortable asking about in a traditional setting [6,34].

To better understand why young women are using TikTok as a source of health information, we asked our respondents about their agreement with various reasons for health-related TikTok use. Our findings showed that the most agreed upon reasons were obtaining advice from others with the same disease or health condition, receiving social support from others, and gaining knowledge about a disease they had been diagnosed with. In an examination of how the current digital landscape has affected Americans’ consumer behavior, CharityRx found “relatability to a shared personal experience” to be a top reason why people go to health influencers for information [12]. In addition to TikTok having the ability to provide immediate information related to users’ health inquiries, it has the capacity to help users locate other people who are similar to them. This may be especially relevant for women. As women have experienced gender bias and poorer treatment in health care settings [35,36], it is possible that they may be especially motivated to seek social support and health information from others like them. Prior research has also indicated that social support is especially beneficial for women [37]. When individuals perceive similarity to a source of information, this can cause the message recipient to feel a stronger sense of connection with the message, which can have important implications in terms of the effects of a message [38]. Perceiving similarity to a source of information can also enhance a user’s perceptions of the message and overall acceptance [39]. In terms of obtaining a further understanding of young women’s motivations for using TikTok as a source of health information, future research could examine what topics (eg, health conditions) young women are most interested in and likely to search for.

Given the potential for misinformation to rapidly pervade the social media landscape, it has been recommended that experts in medical science, public health, and social sciences collaborate to better understand health misinformation on social media, including its reach and influence [40]. Our findings help show the degree to which young women on TikTok perceive an issue of misinformation and to what extent they try to verify or fact-check the information they consume. In first examining the perceived credibility of TikTok health information overall, we found that credibility perceptions were moderate among the respondents who had reported ever having obtained health-related advice or information (intentionally or unintentionally) from TikTok. Notably, health information on TikTok was perceived to be more credible by participants who were older, more educated, or had higher TikTok intensity scores (ie, were more emotionally connected to TikTok and had greater integration of TikTok into their daily lives). While our findings are able to show these positive correlations, we do not know whether the information they are seeing on TikTok (and thus reflecting on when indicating their credibility perceptions) truly is credible. It may be the case that older age, higher levels of education, and more experience with TikTok lead to following more credible users and being delivered more credible content via the TikTok algorithm. In this case, the content may truly be more credible for these users (leading to their greater perceptions of credibility). Future research could explore whether this is the case.

Nearly all participants (1007/1026, 98.15%) indicated that they believe that misinformation is prevalent on TikTok to at least some extent. This may be the result of mainstream news commonly communicating that misinformation is a problem on social media platforms, including TikTok [15]. A great deal of health misinformation reached social media users during the COVID-19 pandemic [8]. It is possible that respondents in this study were among these users or that they heard about this problematic phenomenon. However, despite nearly all participants (1007/1026, 98.15%) stating that they think that misinformation is prevalent on TikTok to at least some extent, only approximately half of the participants (563/1026, 54.87%) indicated that they believe that they have personally encountered health misinformation on TikTok at some point. This discrepancy could stem from a few factors. It could be that the users know that there is a misinformation epidemic but have not been exposed to misinformation because of their commitment to only following credible users (thus leading the TikTok algorithm to feed them more credible content). However, given the large amounts of misinformation that have been identified on the platform [15,41,42] and Americans’ inability to identify most forms of misinformation [43-45], it is more likely that this discrepancy is the result of some respondents not having recognized that they have been exposed to health misinformation. This possibility is further supported by our results that showed that respondents perceive other people as more susceptible to health misinformation on TikTok than they personally are. This finding demonstrates what seems to be a third-person effect, in which the young women perceive that media messages have a greater influence on others than on themselves [23]. As evidence of a third-person effect was provided by the results (with respondents perceiving that other people are more susceptible to health misinformation on TikTok than they personally are), it might be that some of the respondents are naive about their susceptibility to misinformation.

The most common form of verification (to verify the accuracy of the health information found on TikTok) was considering whether the information presented was opinion or fact. The least frequently reported form of verification was verifying the TikTok users’ qualifications or credentials. Age, education, and TikTok intensity were each found to have a weak positive correlation with the likelihood of verifying TikTok health information. Participants who were older, more educated, or had higher TikTok intensity scores were more likely to verify health information on TikTok. As discussed in the Results section, these demographics (age, education, and TikTok intensity) were also found to each be positively correlated with the perceived credibility of TikTok health information overall. Thus, as both credibility perceptions and the verification of the information increase with age, education, and TikTok intensity, perhaps these users truly are seeing more credible content and appropriately perceiving it to be credible. Future research should further explore these relationships because this is beyond the scope of this study. In prior research, fact-checking has typically been found to not be very common on social media. In a survey by Neely et al [16] of Americans’ reliance on social media during the COVID-19 pandemic, it was found that three-fourths of those surveyed had relied on social media to some extent to stay informed about the pandemic, but the majority of them were unlikely to fact-check the information they found.

Finally, this study investigated young women’s perceptions and behaviors related to the top 2 types of sources that share health information on TikTok: health professionals and general users. Prior research has identified that health professionals and general users are 2 of the most prevalent sources on TikTok communicating health information [10,11], and our findings indicate that young women are obtaining health information on TikTok from each of these 2 source types. Of the respondents who had used TikTok, a majority indicated that they had obtained health information both from a health professional on the platform (955/1026, 93.08%) and from a general user on the platform (963/1026, 93.86%). Given that the respondents’ top reasons for health-related TikTok use were obtaining advice from others with the same disease or health condition, receiving social support from others, and gaining knowledge about a disease they had been diagnosed with, it is reasonable that the respondents would perceive both health professionals and general users as valuable sources of information. Prior research exploring the effects of communication sources on social media [9] has explained that both expert-type and peer-type sources provide value. While health professionals have formal training and credentialed experience, general users (eg, peers) can have a form of “experiential credibility” from their own personal experiences (such as that of living with a particular health condition) [9,27]. While most of the respondents had obtained health information from each of the 2 source types, their preference for obtaining health information from a health professional was significantly greater than their preference for obtaining health information from general users (*t*_1025_=23.75; *P*<.001), and, in line with their preferences, the young women reported obtaining health information from health professionals on TikTok significantly more often than they reported obtaining health information from general users on TikTok (*t*_1025_=8.13; *P*<.001). As medical professionals and health institutions delivered COVID-19–related information during the pandemic [5], it was found that Americans who used social media as a source of COVID-19–related information expanded their social media networks to include credible sources (eg, medical institutions and scientific sources) [16]. In addition, CharityRx’s survey found “medical accreditation and certification” to be the top reported reason why people go to influencers for health information [12]. Together, the prior and current findings seem to indicate a preference for obtaining health information from health professionals on platforms such as TikTok. However, it is important to recognize that both of the common source types—health professionals and general users—are providing health information to these users.

Our findings showed that the perceived credibility of TikTok health information from health professionals was significantly higher than the perceived credibility of TikTok health information from general users (*t*_958_=26.737; *P*<.001). This is promising for health professionals who choose to invest in creating a TikTok presence because studies have shown that web-based information is more likely to be attended to when it is perceived as credible [46]. There was also a significant difference in the young women’s likelihood of acting on health information from a health professional versus a general user on TikTok, with their likelihood of acting on the information being greater when the information was from a health professional (*t*_1025_=12.74; *P*<.001). As the respondents had greater perceived credibility of TikTok health information from health professionals (vs general users), it is logical that they would also be more likely to act on health information from health professionals, given that credibility perceptions enhance the likelihood of persuasion [47,48], including in the context of social media messaging [49]. We found that, of the respondents who had received health information from a health professional on TikTok, 43.35% (414/955) reported that they had acted on health information they obtained from a health professional on TikTok. In comparison, 37.8% (364/963) of the respondents who had received health information from a general user on TikTok reported that they had acted on health information they obtained on TikTok from a general user. The respondents’ perceived credibility of health information on TikTok overall was found to be positively correlated with their likelihood of acting on health information from both of the source types, which is in alignment with the relationship between credibility perceptions and persuasive effects [47,48]. On the one hand, these findings are promising in the sense that young women perceive health professionals on TikTok to be more credible and are more influenced by them, further suggesting that it is worthwhile for health professionals to use TikTok as a strategic communication tool. On the other hand, this could mean that young women are more susceptible to being influenced by individuals who give the impression of being qualified health professionals. Medical professionals sometimes provide information that is outside of their scope of expertise [50], and uncredentialed users are often confused for credentialed health professionals [51]. Furthermore, it is important to note that medical credentials and titles vary. A search of the hashtag #womenshealth on TikTok results in videos from a number of different types of health professionals, including nurses, nurse practitioners, obstetrician-gynecologists, medical doctors (with MD or DO credentials), and midwives, and because prior research has shown that many individuals do not understand medical roles and titles or how to differentiate between them [52], this could have profound implications. It is also important to note that anyone on TikTok can present themselves as though they have the necessary credentials for the information they are sharing (eg, adding credentials to their username, presenting themselves with a formal title, wearing a laboratory coat or surgical scrubs, and communicating information in a persuasive manner). An authoritative title, on its own, can be enough to capture an individual’s attention and generate respect [53]. Therefore, with anyone being able to add credentials to their TikTok username, this could be problematic, especially given that credibility is hard to distinguish on social media. Users are more likely to rely on heuristic cues (such as the titles included in a username) to determine a user’s credibility [9,54]. The aforementioned findings also showed that the least frequently reported form of information verification was verifying the TikTok users’ qualifications or credentials, further illustrating that this could be vastly problematic.

The study’s findings showed that the young women in our study are more likely to fact-check information from a general user than fact-check information from a health professional. Again, this is promising in terms of the fact that content from general users may be more likely to include misinformation, but this could be problematic if the credibility cues of a health professional lead users to automatically assume that they can believe and trust any of the information. It would be worthwhile for future research to investigate this further, uncovering whether users trust misinformation from health professionals on TikTok simply because of the creators’ credentials.

We also found that both age and education are positively correlated with young women’s likelihood of acting on health information found on TikTok—both from a health professional and from a general user. However, these correlations were quite weak. A stronger positive correlation was found between the users’ emotional connection to TikTok (ie, TikTok intensity) and their likelihood of acting on health information from both health professionals and general users. It may be logical to assume that users who heavily engage more with TikTok will have a greater propensity to act on the information they receive. Social influence theory suggests that individuals are influenced by those around them [55]. This may extend to the web-based environment, such that as TikTok becomes more integrated into one’s life, it is more likely to affect one’s behaviors.

### Limitations

The findings of this survey research should be interpreted in light of some limitations. First, the sample of survey respondents was recruited through convenience sampling methods. While the sample is only a segment of the total population, we tried to ensure that we had a large sample that was representative of the population of interest (women aged 18-29 years throughout the United States) by using both Qualtrics and 2 large public universities to recruit individuals who fit the sample parameters. This study does not discuss differences between individuals recruited from the universities and those recruited from the Qualtrics sample, but we have provided descriptive statistics for each variable across each sample as a means of allowing for some comparison between the 2 samples (Multimedia Appendix 2). In addition, we made sure to include the respondents’ highest level of education as a variable in this study to see how education is associated with the variables of interest in this study, which can help to provide some understanding of how one’s educational experience might be related to one’s use and perceptions of TikTok health information. Second, because this study relies on self-reporting from the survey respondents, there is a chance that the results do not truly capture the real behaviors of the respondents. As we asked questions about fact-checking web-based information (behaviors that individuals likely know they should engage in), the respondents may have answered some questions in a more socially desirable or acceptable manner (ie, what they expect would be an “appropriate” response) rather than being truthful in their responses. Hopefully, though, because participants knew that their responses would be anonymous, this helped to lessen social desirability bias. Finally, this study focused on young women (assigned female sex at birth) as the population of interest for this study. This narrow focus allows us to better understand the implications of TikTok use for this demographic, but it is also important to explore other populations’ behaviors and perceptions.

### Conclusions and Practical Implications

This study provides a greater understanding of the extent to which TikTok is serving as a source of health information for young women in the United States. With nearly all young women in this study (who had used TikTok) having been exposed to health information on TikTok (948/1026, 92.4%), and more than half of them (582/1026, 56.73%) having actively sought health-related information on the platform in the last 3 months, it is imperative for health professionals and health communication scholars to prioritize the consideration of TikTok as a platform that is influencing health information acquisition and dissemination in the United States. While the popularity and accessibility of TikTok may change, short-form video social media sites are likely to remain a common form of communication [56].

The findings of this study illustrate the potential value that TikTok can have for disseminating health information to an audience of young women. As the respondents of this survey reported a preference for information from health professionals and were more likely to act on the information from these sources, it is worthwhile for health professionals to use TikTok to disseminate health information to this audience, especially given the large number of women on the platform and prior research illustrating that social media significantly influence women’s health-related behaviors and perceptions [17,19]. In doing so, health professionals may want to consider how they can align their content with young women’s most common motivations for using TikTok as a source of health information, which we found to be obtaining advice from others with the same disease or health condition, receiving social support from others, and gaining knowledge about a disease they had been diagnosed with. Given that young women want advice and support from others who are experiencing similar health conditions, it may be useful for health professionals to consider working with individuals who are willing to share their personal experience with a health condition. Incorporating the stories of patients and other experienced individuals who have similar characteristics to those searching for information on TikTok could be especially influential for increasing attention to, and engagement with, health information on TikTok.

Furthermore, given that our findings indicate that young women have a preference for obtaining health information on TikTok from health professionals and that they are less likely to fact-check information from these sources, it is imperative that future initiatives address the proliferation of individuals sharing information beyond their scope of expertise and the problem of social media users confusing uncredentialed users as credentialed health professionals [50,51]. Future researchers and practitioners should also work on media literacy and education initiatives, given the third-person effect found in this research. It seems that young women know that misinformation is an issue on TikTok, but it seems that they may not be recognizing that they have been exposed to misinformation and that they perceive themselves as less susceptible. It may be beneficial for future interventions to address this perception and help young women to have better recognition of when they are being exposed to health misinformation.

## Appendix

Multimedia Appendix 1 Full survey questionnaire.

Multimedia Appendix 2 Descriptive statistics for study measures across 2 samples (student sample and Qualtrics sample).
