# Supplementary material for: TikTok as a Source of Health Information and Misinformation for Young Women in the United States: Survey Study
Source: JMIR Infodemiology. 2024 May 21;4:e54663. doi: 10.2196/54663 (PMC11150891; doi:10.2196/54663)
Supplement: Multimedia Appendix 1 [file infodemiology_v4i1e54663_app1.docx]

**Multimedia Appendix 1: Full Survey Questionnaire**

**Using TikTok as a Health Information Source**

***Frequency of Use***

1. **Have you ever used TikTok**(either to watch videos or post videos)? (Yes/No)
2. On average, **how often do you use TikTok**?
   1. Less than once a month
   2. Once a month
   3. Once a week
   4. A few times a week
   5. Once a day
   6. More often than once a day
3. Have you **ever used TikTok to look for advice or information about your health or health care**? (Yes/No)
4. In the last **3 months,**have you **used TikTok to look for advice or information about your health or health care**? (Yes/No)
5. How often do you **intentionally**use TikTok to get health information or health advice?
   1. Hourly
   2. Daily
   3. Weekly
   4. Monthly
   5. Less often
   6. Not at all
6. How often do you **unintentionally**get health information or health advice from TikTok?
   1. Hourly
   2. Daily
   3. Weekly
   4. Monthly
   5. Less often
   6. Not at all

***Reasons for Health-Related TikTok Use***

1. Please indicate **how much you agree or disagree** with the following statements. (1 = strongly disagree, 7 = strongly agree)
   **I like to get health information from TikTok because:**
   1. It can help me to **maintain a healthy lifestyle**.
   2. It can help me **determine whether I need to see a doctor**.
   3. It can **provide me with more information after I’ve seen my doctor**.
   4. It can help me **find different options for treatment or maintenance of my health condition(s)**.
   5. I can **gain knowledge about a disease I’ve been diagnosed with**.
   6. I can **obtain advice from other patients with the same disease or health condition as me**.
   7. I can **receive social support from others**.
   8. I can **communicate with physicians**.
   9. I can **interact in real time with TikTok users**.
   10. I can **obtain immediate health information and make use of it**.

**Credibility, Misinformation, and Verification of Health Information on TikTok**

***Perceived Credibility of TikTok Health Information Overall***

1. To what degree do you **rate the health information provided on TikTok**?

Not Believable — — — — — — — Believable

Inaccurate — — — — — — — Accurate

Untrustworthy — — — — — — — Trustworthy

Not Biased — — — — — — — Biased

Incomplete — — — — — — — Complete

***Misinformation Perceptions***

1. How prevalent is **health misinformation on TikTok**?

Not at all prevalent — — — — — — — Very prevalent

1. **How serious** do you think the **impact of health misinformation on TikTok** is?

Not at all serious — — — — — — — Very serious

1. **How susceptible are you** to the influence of **health misinformation on TikTok**?

Not at all susceptible — — — — — — — Very susceptible

1. **How susceptible are other people** to the influence of **health misinformation on TikTok**?

Not at all susceptible — — — — — — — Very susceptible

1. Have you **encountered** **health misinformation on TikTok** in the past? (Yes/No)

***Verification of Health Information on TikTok***

1. Please indicate **how often you do the following** when seeing **health information on TikTok.** (1 = never, 7 = always)
   1. Verify the TikTok users’ qualifications or credentials.
   2. Consider the TikTok users’ goals/objectives for posting information online.
   3. Check to see if the information is current.
   4. Seek out other sources to validate the information.
   5. Consider whether the information represented is opinion or fact.
   6. Check to see that the information is complete and comprehensive.

**Health Professionals and General Users as Sources of Health Information on TikTok**

***Source Preferences***

1. Please indicate **how often you get health information** from **health professionals on TikTok.**

Not at all — — — — — — — Very often

1. Please indicate **how often you get health information** from **general users (people like you) on TikTok.**

Not at all — — — — — — — Very often

1. Please indicate **how much you prefer to get health information** from **health professionals on TikTok.**

Don’t prefer them at all — — — — — — — Prefer them a lot

1. Please indicate **how much you prefer to get health information** from **general users (people like you) on TikTok.**

Don’t prefer them at all — — — — — — — Prefer them a lot

***Perceived Credibility of TikTok Health Information from Health Professionals and General Users***

1. To what degree do you **rate the health information** provided by **health professionals** (e.g., a doctor or nurse) on TikTok??

Not Believable — — — — — — — Believable

Inaccurate — — — — — — — Accurate

Untrustworthy — — — — — — — Trustworthy

Not Biased — — — — — — — Biased

Incomplete — — — — — — — Complete

1. To what degree do you **rate the health information** provided by **general users (someone like you**) on TikTok??

Not Believable — — — — — — — Believable

Inaccurate — — — — — — — Accurate

Untrustworthy — — — — — — — Trustworthy

Not Biased — — — — — — — Biased

Incomplete — — — — — — — Complete

***Acting on Health Information***

1. How likely are you to **act on health information** that you see from a **health professional** (e.g., a doctor or nurse) **on TikTok?**

Extremely **unlikely** — — — — — — — Extremely **Likely**

1. How likely are you to **act on health information** that you see from a **health professional** (e.g., a doctor or nurse) **on TikTok?**

Extremely **unlikely** — — — — — — — Extremely **Likely**

1. Have you ever **acted on health information** you got from a **health professional** (e.g., a doctor or nurse) **on TikTok**? (Yes/No)
2. Have you ever **acted on health information** you got from a **general user** (someone like you) **on TikTok**? (Yes/No)

***Fact-Checking Information***

1. How likely are you to **fact-check** health information**from a health professional**(e.g., a doctor or nurse) on TikTok?

Not at all likely — — — — — — — Very **Likely**

1. How likely are you to **fact-check** health information**from a general user**(e.g., someone like you) on TikTok?

Not at all likely — — — — — — — Very **Likely**

**Audience Characteristics**

***TikTok Intensity***

1. Please indicate **how much you agree or disagree** with the following statements. (1 = strongly disagree, 7 = strongly agree)
   1. TikTok is part of my everyday life.
   2. I am proud to tell people I’m on TikTok.
   3. TikTok has become part of my daily routine.
   4. I feel out of touch when I haven’t logged into TikTok for a while.
   5. I feel I am part of the TikTok community.
   6. I would be sorry if TikTok shut down.

**Demographic Questions**

1. What is your age? (Please type your age in years.)
2. What most closely aligns with your **biological sex at birth**?
   1. Male
   2. Female
   3. Intersex
   4. Prefer not to say
3. What is your **gender identity**?
   1. Man
   2. Woman
   3. Trans Man
   4. Trans Woman
   5. Nonbinary
   6. Gender Fluid
   7. Other
4. What is your **race?**
   1. White
   2. Black or African American
   3. American Indian or Alaska Native
   4. Asian
   5. Native Hawaiian or Pacific Islander
   6. Other
   7. Prefer not to answer
5. What is your **highest level of education?**
   1. Less than high school
   2. High school graduate or equivalent (e.g., GED)
   3. Some college
   4. 2-year degree
   5. 4-year degree
   6. Professional or master’s degree
   7. Doctorate
