# Supplementary material for: TikTok as a Source of Health Information and Misinformation for Young Women in the United States: Survey Study
Source: JMIR Infodemiology. 2024 May 21;4:e54663. doi: 10.2196/54663 (PMC11150891; doi:10.2196/54663)
Supplement: Multimedia Appendix 2 [file infodemiology_v4i1e54663_app2.docx]

**Multimedia Appendix 2: Descriptive Statistics for Study Measures Across Two Samples (Student Sample and Qualtrics Sample)**

# Descriptive Statistics for Study Measures Across Two Samples (Student Sample and Qualtrics Sample)

| **Supplementary Table 1.** Frequency of TikTok Use across Student Sample and Qualtrics Sample | | | |
| --- | --- | --- | --- |
| Question and responses | | Student Sample  *n* (%) | Qualtrics Sample  *n* (%) |
| **Ever Used TikTok** | | |  |
|  | Yes | 526 (98.13%) | 500 (78.62%) |
|  | No | 10 (1.87%) | 136 (21.38%) |
| **Average TikTok Use (Among those who have used TikTok)** | | |  |
|  | Less than once a month | 28 (5.32%) | 30 (6.00%) |
|  | Once a month | 9 (1.71%) | 41 (8.20%) |
|  | Once a week | 9 (1.71%) | 51 (10.20%) |
|  | A few times a week | 36 (6.84%) | 72 (14.40%) |
|  | Once a day | 62 (11.79%) | 73 (14.60%) |
|  | More often than once a day | 382 (72.62%) | 233 (46.60%) |
| **Ever intentionally used TikTok to look for advice or information about health or health care** | | |  |
|  | Yes | 325 (60.63%) | 347 (54.56%) |
|  | No | 211 (39.37%) | 289 (45.44%) |

| **Supplementary Table 2.** Frequency of Using TikTok Intentionally and Unintentionally as a Source of Health Info Among Respondents Who Had Ever Used TikTok across Student Sample and Qualtrics Sample | | | |
| --- | --- | --- | --- |
| Question and responses | | Student Sample  *n* (%) | Qualtrics Sample  *n* (%) |
| **Frequency of Intentional Use of TikTok to Get Health Information** | | |  |
|  | Hourly | 5 (.95%) | 24 (4.80%) |
|  | Daily | 5 (.95%) | 103 (20.6%) |
|  | Weekly | 33 (6.27%) | 110 (22.00%) |
|  | Monthly | 112 (21.29%) | 56 (11.20%) |
|  | Less often | 147 (27.95%) | 43 (8.60%) |
|  | Not at all | 224 (42.59%) | 164 (32.80%) |
| **Frequency of Unintentional Exposure to Health Information on TikTok** | | |  |
|  | Hourly | 4 (.76%) | 34 (6.80%) |
|  | Daily | 73 (13.88%) | 159 (31.80%) |
|  | Weekly | 231 (43.92%) | 132 (26.40%) |
|  | Monthly | 111 (21.10%) | 61 (12.20%) |
|  | Less often | 70 (13.31%) | 66 (13.20%) |
|  | Not at all | 37 (7.03%) | 48 (9.60%) |

| **Supplementary Table 3.** Agreement with Reasons for Health-Related TikTok Use Among Respondents Who Had Ever Used TikTok across Student Sample and Qualtrics Sample | | | |
| --- | --- | --- | --- |
| Reason | | Student Sample  Level of Agreement, *M*(*SD*) | Qualtrics Sample  Level of Agreement,  *M*(*SD*) |
| **I like to get health information from TikTok because…** | |  |  |
|  | It can help me to maintain a healthy lifestyle. | 4.91 (1.55) | 4.94 (1.65) |
|  | It can help me determine whether I need to see a doctor. | 4.65 (1.69) | 4.98 (1.62) |
|  | It can provide me with more information after I've seen my doctor. | 4.41 (1.71) | 4.90 (1.69) |
|  | It can help me find different options for treatment or maintenance of my health condition(s). | 4.56 (1.69) | 5.04 (1.58) |
|  | I can gain knowledge about a disease I've been diagnosed with. | 4.82 (1.66) | 5.20 (1.49) |
|  | I can obtain advice from other patients with the same disease or health condition as me. | 5.32 (1.56) | 5.25 (1.53) |
|  | I can receive social support from others. | 5.19 (1.65) | 5.40 (1.47) |
|  | I can communicate with physicians. | 3.40 (1.76) | 4.76 (1.71) |
|  | I can interact in real time with TikTok users. | 4.11 (1.84) | 5.09 (1.63) |
|  | I can obtain immediate health information and make use of it. | 4.13 (1.71) | 4.96 (1.61) |
| *Note.* Respondents indicated level of agreement using a 7-point scale (1 = strongly disagree, 7 = strongly agree). | | | |

| **Supplementary Table 4.** Perceived Credibility of TikTok Health Information across Student Sample and Qualtrics Sample | | |
| --- | --- | --- |
| Question | Student Sample  *M*(*SD*) | Qualtrics Sample  *M*(*SD*) |
| Perceived Credibility of TikTok Health Information *Overall* | 4.03 (1.16) | 5.00 (1.51) |
| Perceived Credibility of TikTok Health Information From *Health Professionals* | 5.02 (1.19) | 5.32 (1.23) |
| Perceived Credibility of TikTok Health Information From *General Users* | 3.27 (1.35) | 4.68 (1.40) |

*Note.* Five items (each measured using a 7-point bipolar scale) were averaged to create perceived credibility scores.

| **Supplementary Table 5.** Perceived Prevalence, Seriousness, and Susceptibility of Health Misinformation on TikTok Among Respondents Who Had Ever Used TikTok across Student Sample and Qualtrics Sample | | |
| --- | --- | --- |
| Question | Student Sample  *M*(*SD*) | Qualtrics Sample  *M*(*SD*) |
| How prevalent is health misinformation on TikTok? | 5.28 (1.33) | 5.00 (1.51) |
| How serious do you think the impact of health misinformation on TikTok is? | 5.80 (1.30) | 5.32 (1.59) |
| How susceptible are you to the influence of health misinformation on TikTok? | 4.10 (1.69) | 4.04 (1.81) |
| How susceptible are other people to the influence of health misinformation on TikTok? | 5.51 (1.30) | 4.99 (1.57) |
| *Note.* Respondents indicated their responses using a 7-point scale (1 = not at all prevalent/serious/susceptible, 7 = very prevalent/serious/susceptible). | | |

| **Supplementary Table 6.** Source Preferences and Fact Checking Across Sources across Student Sample and Qualtrics Sample | | |
| --- | --- | --- |
| Question | Student Sample  *M*(*SD*) | Qualtrics Sample  *M*(*SD*) |
| Please indicate how often you get health information from health professionals on TikTok. | 5.00 (1.78) | 5.09 (1.88) |
| Please indicate how often you get health information from general users (someone like you) on TikTok. | 4.42 (1.92) | 4.68 (1.86) |
| Please indicate how much you prefer to get health information from health professionals on TikTok. | 5.82 (1.77) | 5.47 (1.71) |
| Please indicate how much you prefer to get health information from general users (someone like you) on TikTok. | 3.66 (2.01) | 4.52 (1.80) |
| How likely are you to fact-check health information from a health professional on TikTok? | 4.54 (1.85) | 5.25 (1.68) |
| How likely are you to fact-check health information from a general user (e.g., someone like you) on TikTok? | 5.30 (1.98) | 5.45 (1.65) |
| *Note.* Respondents indicated their responses using a 7-point scale (1 = not at all/don’t prefer them at all/not at all likely, 7 = very often/prefer them a lot/very likely). | | |

| **Supplementary Table 7.** Likelihood of acting on Health Information across Student Sample and Qualtrics Sample | | |
| --- | --- | --- |
| Question | Student Sample  *M*(*SD*) | Qualtrics Sample  *M*(*SD*) |
| How likely are you to act on health information that you see from a health professional (e.g., a doctor or nurse) on TikTok? | 4.16 (1.80) | 4.87 (1.72) |
| How likely are you to act on health information that you see from a general user (e.g., someone like you) on TikTok? | 3.33 (1.76) | 4.62 (1.81) |
| *Note.* Respondents indicated their likelihood using a 7-point scale (1 = extremely unlikely , 7 = extremely likely). | | |

| **Supplementary Table 8.** Number of TikTok users who have Acted on Health Information from Health Professionals and General Users across Student Sample and Qualtrics Sample | | |
| --- | --- | --- |
|  | Student Sample  *n* (*%*) | Qualtrics Sample  *n* (*%*) |
| Has acted on TikTok Health Information From a Health Professional | 146 (27.81%) | 268 (53.60%) |
| Has acted on TikTok Health Information From a General User | 121 (23.05%) | 243 (48.60%) |
|  | | |

| **Supplementary Table 9.** Verification Behaviors for TikTok Health Information Among Participants Who Had Intentional or Unintentional Exposure to Health Information on TikTok across Student Sample and Qualtrics Sample | | |
| --- | --- | --- |
| Verification Behavior | Student Sample  *M*(*SD*) | Qualtrics Sample  *M*(*SD*) |
| Verify the TikTok users' qualifications or credentials. | 4.12 (1.98) | 4.95 (1.86) |
| Consider the TikTok users’ goals/objectives for posting information online. | 4.32 (1.87) | 4.97 (1.66) |
| Check to see if the information is current. | 4.42 (1.86) | 5.29 (1.59) |
| Seek out other sources to validate the information. | 4.68 (1.86) | 5.32 (1.62) |
| Consider whether the information represented is opinion or fact. | 4.86 (1.84) | 5.43 (1.49) |
| Check to see that the information is complete and comprehensive. | 4.40 (1.84) | 5.41 (1.57) |

*Note.* Respondents indicated how often they do each verification behavior using a 7-point scale (1 = never , 7 = always).

| **Supplementary Table 10.** TikTok Intensity Scores across Student Sample and Qualtrics Sample | |
| --- | --- |
|  | *M* (*SD*) |
| Student Sample | 4.82 (1.47) |
| Qualtrics Sample | 5.00 (1.51) |

*Note.* Six items (each measured using a 7-point bipolar scale) were averaged to create TikTok intensity scores.
